# Stereotactic radiotherapy in epithelial ovarian cancer brain metastases patients

**DOI:** 10.1186/s13048-014-0079-1

**Published:** 2014-08-15

**Authors:** Agata Celejewska, Andrzej Tukiendorf, Leszek Miszczyk, Krzysztof Składowski, Jerzy Wydmański, Krystyna Trela-Janus

**Affiliations:** Radiotherapy Department, Cancer Center and Institute of Oncology, 15 Wybrzeże AK Str., 44-101 Gliwice, Poland; Department of Epidemiology, Cancer Center and Institute of Oncology, 15 Wybrzeże AK Str., 44-101 Gliwice, Poland; Radiotherapy and Chemotherapy Clinic, Cancer Center and Institute of Oncology, 15 Wybrzeże AK Str., 44-101 Gliwice, Poland

**Keywords:** Epithelial ovarian cancer, Brain metastases, Stereotactic radiotherapy, Risk factors, Survival analysis, Classification tree

## Abstract

**Background:**

In this report we present the results of the retrospective (survival and classification) analyses of possible prognostic factors prolonging survival in epithelial ovarian cancer brain metastases patients after stereotactic radiotherapy. We focus on a wide range of available predictors to establish survival in patients with a good health status and no more than three lesions.

**Methods:**

Two parallel statistical methods in survival analysis were used: classical and Bayesian methods to verify statistical results. To display the predicted and posterior survivals, classification trees were built.

**Results:**

From the initial set of prognostic factors, only four were established as statistically significant in multivariate regression. They were: survival to metastases to brain after epithelial ovarian cancer diagnosis, number of metastases at diagnosis, central nervous system radiotherapy prior to stereotactic radiotherapy, and interval to stereotactic radiotherapy after metastases diagnosis.

**Conclusions:**

When considering evidence-based standards of treatment of patients suffering from epithelial ovarian cancer brain metastases, the established clinical factors are suggested to be prognostic.

## Background

Brain metastases (BMs) from epithelial ovarian cancer (EOC) are rare (approximately from one to a several percent [1–5]), but diagnosis of this occurrence is increasing in recent years [6–8], probably owing to more effective treatment of the primary cancer and the resulting prolongation of survival [2]. However, the prognosis for patients with BM from EOC is poor [9–11,4].

In the last few decades many investigations have been carried out to gain a better understanding of the mechanism of ovarian cancer brain dissemination and different factors have been established but partly with conflicting results [4]. However, due to the small number of patients considered in these studies [12–14], randomized treatment evaluations specific for this histology are not possible [2]. Therefore, in order to critically evaluate underlying risk factors and the clear benefit of treatment options, multicenter clinical trials are needed in further investigation [9].

Only two factors were able to gain acceptance of most authors publishing in the current literature on the subject [4]. One factor is the presence of an extracranial disease at the time of brain relapse, which was shown to have a significantly negative impact on survival. The other factor is the performance status of patients at the time of recurrent disease [2,4]. Moreover, a short time interval between primary diagnosis and the brain relapse was reported to have a negative impact on survival [4]. An alternative univariate analysis has shown that extraperitoneal metastases at the time of the diagnosis of brain metastases are adversely correlated with survival [2] (these results comply with those of [13]). Some authors report that the Fédération Internationale de Gynécologie et d’Obstétrique (FIGO) stage has been correlated with increased incidence of brain metastasis [12,15].

In the case of other factors which were investigated in the past, such as age at diagnosis, time interval between primary diagnosis and metastatic manifestation, number of BMs, tumor stage, grade, histotype, degree of histological differentiation and site of lesion of central nervous system (CNS) relapse, it is widely agreed in the current literature that these are not related to survival [4,10,13,9,16]. What is more, CA125, a useful biomarker for detecting relapse of ovarian cancer, has a limited value in detection of brain metastases [17–19,5] (theoretically, because the blood–brain barrier may hinder the relatively large CA125 molecule produced by cerebral lesions from gaining access to systemic blood circulation [5]).

Considering that brain dissemination in ovarian cancer is generally a palliative situation, quality of life should be one of the main objectives that has to be taken into account in the decision on the optimal therapy strategy. All the patients report discomfort after treatment [4], and no definite therapeutic modality can be inferred [5]. A multi-modal approach using surgery, radiation and chemotherapy (CT) has been suggested in several reports. The standard treatment consists of full brain radiotherapy (WBRT), which was shown to improve the quality of life by reduction of neurological symptoms and appears to result in a prolongation of survival [18,10]. There was a statistically significant difference in survival for those treated with WBRT plus CT versus those treated with WBRT alone, and those undergoing supportive care only [3]. However, most CT related side-effects result in a decrease of a patient’s quality of life.

In the last few years stereotactic radiosurgery (SRS – with a single fraction) as well as stereotactic radiotherapy (SRT – with a high-dose radiation fraction) have come into focus as other promising therapy options in brain metastases from ovarian cancer [4] (in our paper both defined as SRT). Some authors [11] even describe the observed remarkable median survival after treatment with SRS as compared to WBRT. Indeed, SRT is now considered by many groups to be an alternative to surgery for focal control of cerebral metastases in certain situations, and has become increasingly popular [20] as another promising therapy option or even optimal treatment [21,11,22]. The presented radiotherapeutic direction is consistent with that presumed in the initial report on the EOC BM patients advocating that “*prompt aggressive therapy of the CNS metastasis may provide not only adequate palliation, but prolonged survival*” [1].

In the present study, we reviewed our experience with EOC BM patients who underwent SRT. The aim of the study was to evaluate the efficacy of the EOC BM treatment in patients after SRT and to investigate possible prognostic factors of survival.

## Material and methods

The analyzed material (with obtained consent) comprised of 32 patients (in comparison with 23 in [13], or 12 in [20]) who were diagnosed with BM from EOC and underwent SRT in the Cancer Center and Institute of Oncology in Gliwice, Poland, between 2003 and 2013 (with a prior EOC diagnosis since 1998). General characteristics of patients (with basic description statistics) classified in the following groups of risk factors are: age, clinical, pathological, imaging, treatment, and follow-up outcome, as presented in Table 1.

**Table 1:** **Data on EOC BM patients**

**Characteristics**	**Risk factor**	**Mean**	**SD**	**Median**	**Q1**	**Q3**	**Min.**	**Max.**
Age	at EOC diagnosis	52.6	9.3	54	47	59.3	27	67
	at SRT	56.3	9.2	56.5	50.8	62	35	77
Clinical	CA125 before CT1 [U/mL]	1578	2317	740	151	1946	19	9693
	CA125 after CT1 [U/mL]	29	69	13	8	17	2	318
	CA125 before CT2 [U/mL]	566	791	190	84	690	38	2407
	CA125 after CT2 [U/mL]	73	143	22	10	57	9	496
	CA125 before SRT [U/mL]	132	189	56	25	139	8	533
	ZUBROD	1.2	0.4	1	1	1	1	3
	FIGO			IIIC	IIIA	IIIC	IA	IVA
	Tumor grade [G]	2.6	0.5	3	2	3	2	3
	Histopathology:	*adenocarcinoma serosum* = 53%, *adenocarcinoma endometroides* = 25%, *cystadenocarcinoma mucinosum* = 6%, non-differentiated = 13%, not examined = 3%						
Imaging	No. of BMs at diagnosis	1.8	0.8	2	1	2	1	3
	Total PTV at diagnosis [cm^3^]	9.7	14.8	5.1	1.6	10.0	0.4	62.1
	Relapse out of CNS	47%						
	Localisation	supratentorial = 25%, infratentorial = 63%, supratentorial/infratentorial = 12%						
Treatment	from EOC diagnosis to CT1 [weeks]	6.6	5.9	5	3	8	0	28
	CT1 duration [weeks]	19.6	5.6	18	17	20	12	38
	CT2 duration [weeks]	8.5	10.4	0	0	17	0	34
	Total CT duration (CT1 + CT2) [weeks]	27.7	13	23	18	35	12	72
	No. of CT1 cycles	6.1	0.8	6	6	6	5	9
	No. of CT2 cycles	2.4	2.9	0	0	5.5	0	9
	Total no. of CT cycles (CT1 + CT2)	8.5	3.2	6	6	11.5	5	18
	Surgery	22%						
	WBRT before SRT	53%						
	WBRT after SRT	16%						
	BM to SRT [months]	22	26.9	8	4.8	36	1	117
	BM to SRT (above 1 month)	75%						
Follow-up outcome	from EOC diagnosis to BM [months]			29.5(CI95% = 25–44)				
	from BM to death [months]			16(CI95% = 8–21)				
	from SRS to death [months]			7(CI95% = 6–18)				
	from EOC diagnosis to death [months]			49(CI95% = 45–69)				
	Deaths during observation	94%						

Altogether, over 30 continuous, binary, ordered, and categorical variables were taken into account (see Table 1).

In particular, wide ranges of CA125 protein concentrations were observed before CT, while their levels decreased after treatment. 28 of 32 patients were classified by the FIGO staging of ovarian carcinomas (17 cases with IIIC stage), and in 26 of those the tumor differentiation was examined (16 patients had prevailingly G3 grading).

Imaging analyses were conducted in all patients (16 following computer tomography, and the remaining half following magnetic resonance). Of those, in 14 patients a single BM was detected, 12 of them had 2 BMs, and 6 patients were diagnosed with 3 BMs.

With respect to RT, after BM diagnosis, 23 patients were irradiated with SRS dose of 8–24 Gy, and 9 of them underwent SRT in 2–3 fractions of 12–24 Gy doses. A large number of patients (22) received WBRT with the most frequent fractionation schedule 5 × 4 Gy (17 of them had WBRT prior to SRT, and 5 post-SRT). Prior to SRT, 5 patients had also undergone surgical resection.

**Figure 1:**
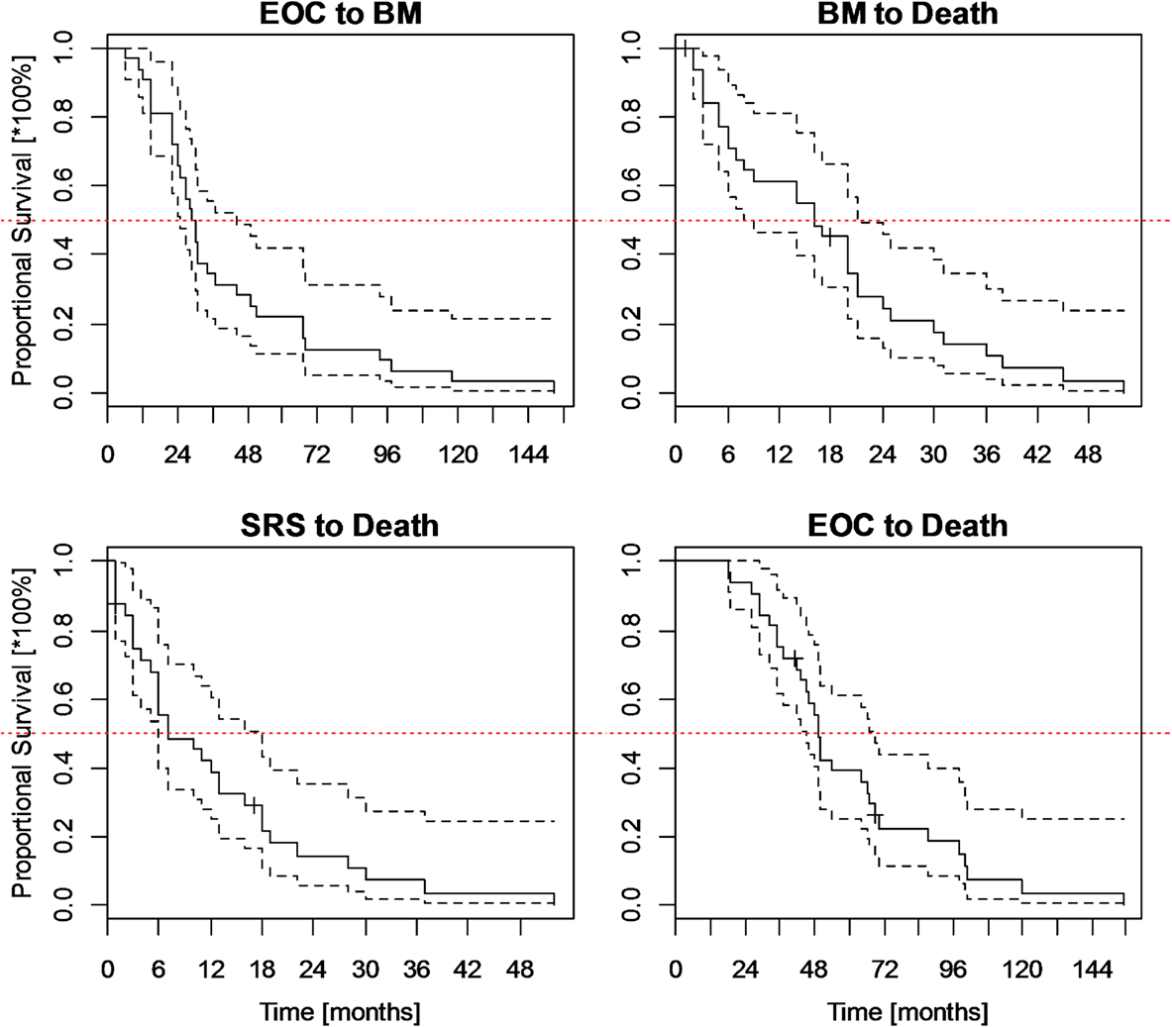
**Survival in EOC BM patients.**

The Kaplan-Meier survival curves in a combined plot are presented in Figure 1.

In comparison, according to the different types of lesions, the median of BM free survival (BMFS) = 15.9 months was observed in Greek patients [3], 22 months in a Taiwanese study [8], and 57 months in patients with grade I and II tumors in an Israeli investigation [2]. In turn, the post-BM survival ranged from 2 months in patients on corticosteroids [2], to 29 months in patients after treatment with SRT [11].

Relationships between possible risk factors and survival after SRT were tested using available modern statistical methodology. In the survival analysis, the Weibull’s regression [23] has been applied. The results were presented as hazard ratios with 95% confidence/credible intervals following both classical (frequentist) [24] and Bayesian (simulation) approaches [25].

Subsequently, the respective predicted and posterior survivals from classical and Bayesian models, were used to build classification trees of patients [26]. In the analysis, “rpart” statistical package [27] of the R software [24] was implemented.

## Results

The results of multivariate analyses of survival after SRT are reported in Table 2.

**Table 2:** **Multivariate Weibull’s regression**

**Method:**	**Classical**			**Bayesian**		
**Risk factor**	**Hazard ratio**	**Confidence 95% interval**	***p*** **-Value**	**Hazard ratio**	**Credible 95% interval**	***p*** **-Value (one-sided)**
Time from EOC diagnosis to BM	1.022	(1.007,1.039)	0.0051	1.022	(1.005,1.037)	0.0054
Number of BMs	1.931	(1.202,3.102)	0.0065	1.999	(1.150,3.266)	0.006
WBRT prior to SRT	0.211	(0.07,0.635)	0.0057	0.249	(0.065,0.674)	0.0054
Interval to SRT longer than 1 month vs. shorter than 1 month	20.06	(6.016,66.91)	<0.0001	28.02	(5.003,88.83)	<0.0001

**Figure 2:**
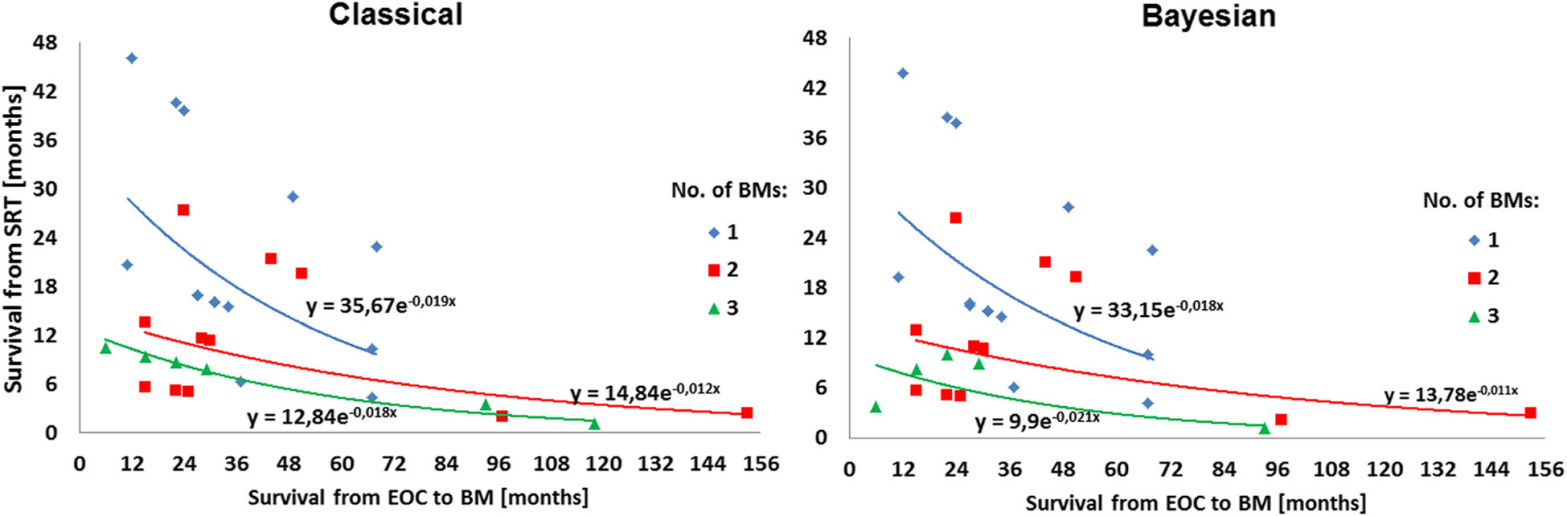
**Median survival since SRT vs. time from EOC to BM stratified by BM numbers.**

**Figure 3:**
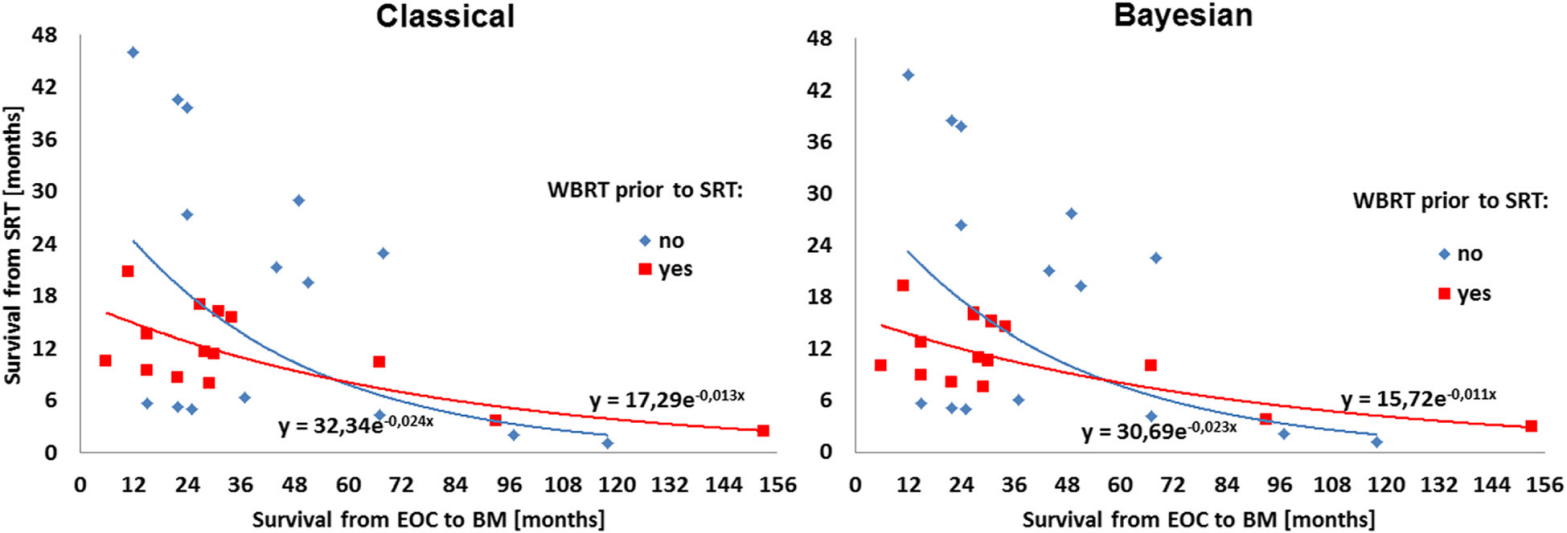
**Median survival since SRT vs. time from EOC to BM stratified by prior WBRT.**

**Figure 4:**
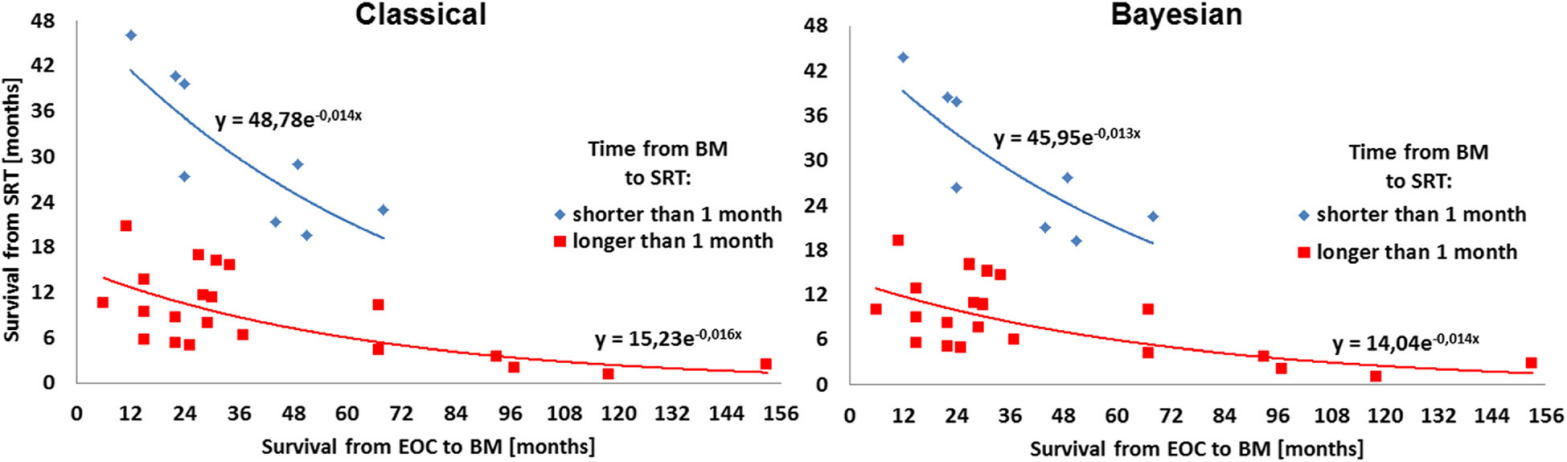
**Median survival since SRT vs. time from EOC to BM stratified by interval to SRT.**

In the multivariate regression analysis, only four possible risk factors were found that might have an influence on the risk of early death after EOC BM diagnosis in the analyzed group of patients and, worthy of note, they are nearly identical both in the classical and Bayesian approaches (see Table 2). Interpretation of the results is as follows: each additional month of BMFS resulted in the 2% increase of risk of death in patients after a disease metastasis; if the difference was one year, then the risk increased up to (1.022^12^–1)*100% = 30%. The number of metastases may also elevate the risk of death in EOC patients: in those with 2 BMs, the risk is nearly twice as high compared with patients suffering from 1 BM only, according to both classical and Bayesian approaches; whereas in patients with 3 BMs the risk was approximately 4 times higher than in those with 1 BM after the metastasis. WBRT applied prior to SRT significantly delayed death of patients after BM; the risk of early death was reduced by over ¾. Worthy of note, delayed SRT, longer than 1 month after BM diagnosis, drastically (at least 20 times) increased the risk of death in EOC patients. The obtained relationships for predicted outcomes in different dimensions (with exponential approximations) for classical and Bayesian approaches are presented graphically in Figures 2, 3 and 4.

It can be seen in Figures 2, 3 and 4 that SRT considerably increased median survival in EOC BM patients, however, a reducing effect can be observed with time to metastasis. The best prognosis is expected for patients with a single BM, whereas in those with 2 or 3 lesions, the survival after the SRT treatment decreases radically. Following the models, in patients who had BMFS for approximately 5 yrs., a beneficial effect of WBRT prior to SRT for survival was observed. However, reduced time to SRT after BM diagnosis is the strongest argument for the survival prolongation. Finally, based on the Weibull’s regression parameters strong coherence between the classical and Bayesian methods can be established.

**Figure 5:**
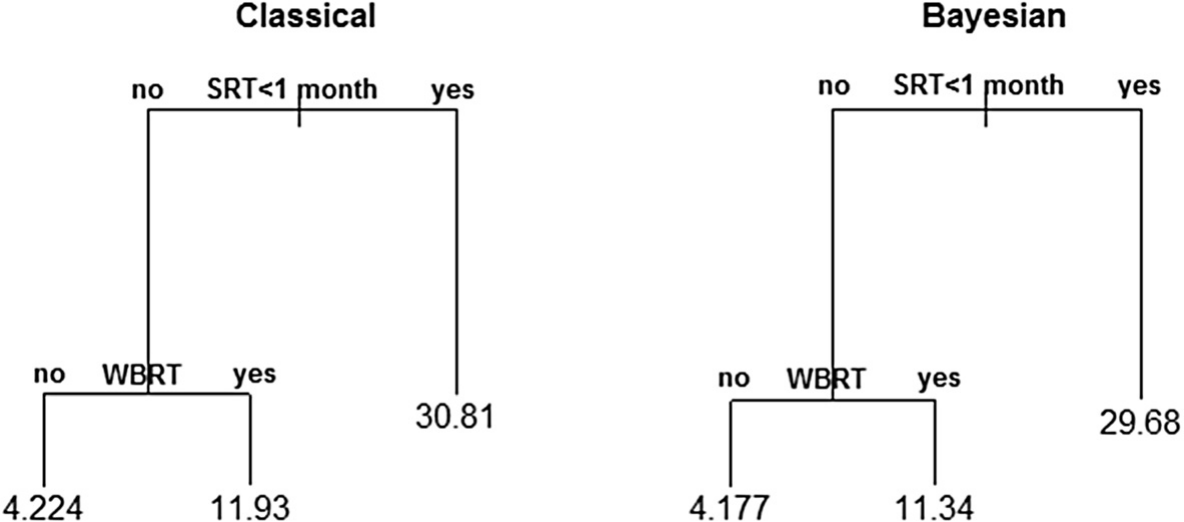
**Classification trees of the classical and Bayesian median survivals.**

The plots of classification trees of the predicted and posterior survivals in EOC BM patients after SRT for the classical and Bayesian approaches are presented in Figure 5.

Following classification trees (Figure 5), it can be ascertained that interval to SRT after metastasis is also a basic factor for the modeled (classical and Bayesian) survivals; prompt SRT provided the longest median survival reaching approximately 30 months. In case of longer expectation for SRT (>1 month), one-year survival with a prior WBRT is predicted. The poorest (four-month) survivals were observed in patients with longer interval for SRT and with no prior WBRT.

## Discussion

Defining possible clinical and medical factors is a very difficult research task, so is standardization of treatment prolonging survival in EOC BM patients. Contemporary efforts show different research outcomes but they are not much consistent in worked conclusions. What is more, already established prognostic factors often did not pass the multivariate analysis [13,18,4]. The reason for such situation is a clinical specificity and epidemiological rarity of the disease. Our results based on the multivariate regression confirm previous reports and show some novelty.

Disputable appears to be an inverse correlation between BMFS and survival after SRT. Our result is opposite to [13] who report a median survival of 9 months in patients with BMFS < 40 months, and 16 months after SRT, if BMFS > 40 months. There are two possible explanations for longer survival after SRS/SRT and short BMFS. What is important, brain metastases are a crucial factor influencing survival of ovarian cancer patients, however not the only one. Clinical situation of solitary brain metastasis as only one location of cancer is extremely rare and possibility of presence of other metastases increases with time after the basic treatment completion. In a disseminated disease also the other metastases limit survival, what could partially explain this situation. The second reason for such correlation can be a delay in brain metastases diagnosis, what is linked to their volume and to limited survival (longer time to diagnosis – larger tumor – shorter survival).

The number of lesions to the brain is another most probable risk factor of survival. Our result is consistent with [18] who describes a slight advantage of a solitary lesion over presentation with multiple lesions (with a median survival of 7 versus 5 months at a liberal *p*-Value = 0.07 [18]). This result is in an absolute agreement with logic and neoplastic disease biology. More metastases (more cancer clonogenic cells) is always connected to shortened survival; especially in the case of brain mets located in a limited volume of skull. Exactly the same results are obtained in other cases of cancers.

A novel finding of our study is WBRT before SRT, which has never been reported so far in case of EOC BM patients (although it’s been already established for other primary tumor sites [28]). We estimated a beneficial effect of preceding WBRT in patients with BMFS > 5 years (post-SRT WBRT did not bring any statistical effects on survival). This result could be biased by a retrospective character of this study and patient selection. Probably, a greater number of ovarian cancer patients were treated in this period with WBRT because of brain mets; but only few, very well responding, had following SRS/SRT. Good individual response to radiation probably impacted the survival. Additionally, late presence of BM, can be linked to a lesser aggressiveness of the (individual) disease itself.

Since publication of the original study [1], most of the reports support the thesis of a prompt aggressive radiotherapy as very promising in the disease treatment. We do support this opinion but SRT must be conducted immediately after BM diagnosis (<1 month). Then, it may considerably affect the disease development and extend life expectancy. Because a remarkable median survival of 29 months after SRT compared to 6 months after WBRT was observed in [11], we suggest to use the RT techniques together, as opposed to SRT alone. As aforementioned, the impact of WBRT-SRS/SRT combination could be influenced by patient selection and individual features of particular cancers, however, considering a systemic character of ovarian cancer (similarly as in, for example, small cell lung cancer), we can expect better results of combined RT modality with a relatively short gap (elective effect of WBRT + local additive effect of WBRT/SRT combination).

In classification analysis, the applied statistical algorithm has “chosen” RT schedules (expectation interval for SRT and prior WBRT in the order of importance) as more effective than other, found in this study, risk factors (BMFS and number of BMs). This fact magnifies medical supremacy of radiotherapy in cancer treatment.

## Conclusions

Based on the experience from Cancer Center and Institute of Oncology in Gliwice, we can draw the following conclusions:

- prompt SRT may prolong survival in EOC BM patients;
- additional prior WBRT is suggested in patients who had BMFS > 5 years;
- BMFS and number of BMs are reliable prognostic factors of survival;
- multiclinic trials and cooperation in the future may extend the medical knowledge on the considered problem.
